# Fungal Immune Evasion in a Model Host–Pathogen Interaction: *Candida albicans* Versus Macrophages

**DOI:** 10.1371/journal.ppat.1003741

**Published:** 2013-11-21

**Authors:** Claudia Jiménez-López, Michael C. Lorenz

**Affiliations:** Department of Microbiology and Molecular Genetics and the Graduate School of Biomedical Sciences, the University of Texas Health Science Center, Houston, Texas, United States of America; The University of North Carolina at Chapel Hill, United States of America

The innate immune system is the primary line of defense against systemic fungal infections. As a result, the interaction between *Candida albicans*, the most important fungal pathogen in the developed world, and innate immune phagocytes has received significant attention as a key infection determinant, revealing complex transcriptional and developmental responses in both cell types. The availability of easily propagated macrophage-like cell lines and the remarkable morphological switch that occurs in phagocytosed *C. albicans* cells have made this an important model of host–pathogen interactions. Several lines of evidence have emerged that *C. albicans* actively resists recognition by the immune system and inhibits some of the classical antimicrobial responses. This Pearl will discuss recent findings in *Candida*–macrophage interactions.

## Immune Recognition: A Taste of Something Sweet

Innate immune phagocytes, including macrophages, recognize *C. albicans* and other fungal pathogens via Pathogen Associated Molecular Patterns (PAMPs); the most important of these are the cell wall carbohydrates: mannan (as mannosylated proteins), β-glucan, and chitin, a minor component. The structure of these polysaccharides and the receptors to which they bind has been discussed in depth elsewhere [1], [2]. In *C. albicans* yeast cells, the β-glucan layer is obscured by the outer mannoproteins and largely inaccessible to the immune system [3]. To oversimplify, a greater proinflammatory response results from alterations that increase exposure of the inner β-glucans, including hypoxia [4], antifungal drugs [3], and inhibition of glycerophosphatidylinositol (GPI)-anchoring of the mannoproteins in the wall [5]. Some strain-specific differences in β-glucan–based recognition by the Dectin-1 receptor are apparent only in vivo but affect the outcome of the infection [4]; one could speculate that this represents active regulation of cell wall structure in vivo.

Deposition of complement proteins on the cell surface is both microbicidal and immunostimulatory. The *C. albicans* cell surface binds numerous negative regulators of the complement cascade to inhibit complement activation: Pra1 (pH-Regulated Antigen) binds plasminogen, Factor H, FHL-1, and C4B, all in active forms [6], [7]. At least eight proteins bind plasminogen to the *C. albicans* surface [8], and complement factors are substrates of the Secreted Aspartyl Protease family [9]. The sum of these activities is to reduce immune recognition of the fungal cell.

## Stress Responses: Pleading Self-Defense

*C. albicans* is resistant to macrophage-associated stresses, including reactive oxygen and nitrogen species (ROS/RNS). *C. albicans* encodes a catalase and six superoxide dismutases; unusually, three Sod enzymes (Sod4-6) are secreted and detoxify extracellular ROS produced by macrophages [10]. The single catalase (Cat1) does not have a signal sequence but has also been identified on the cell surface [8]. Thus, *C. albicans* blunts the antimicrobial respiratory burst before it can cause intracellular damage. Anti-RNS defenses are intracellular in the form of three flavohemoglobin enzymes (Yhb1, Yhb4, Yhb5); deletion of *YHB1* renders cells hypersensitive to NO in vitro [11]. There is also evidence that a variety of fungi, including *C. albicans*, can inhibit NO production from macrophages, but no mechanism has been identified (see [12]).

A discrepancy exists, however, between in vitro and in vivo stress phenotypes. Mutation of the ROS-responsive Cap1 transcription factor confers profound sensitivity to oxidants and failure to filament within macrophages [13], [14]. Cap1 regulates catalase expression [15], as does the MAP kinase Hog1; both *cat1*Δ and *hog1*Δ have profound virulence defects in mice [16], [17]. Yet the *cap1*Δ mutant is fully virulent [18], suggesting additional Hog1- and Cap1-independent regulatory mechanisms, as proposed [19]. Similarly, mutation of Yhb1 has only mild effects on virulence, suggesting roles for other anti-NO mechanisms in vivo [11].

## Nutritional Stress: The South Beach Diet

*C. albicans* cells phagocytosed by macrophages switch to a gluconeogenic growth mode [20]. The starvation-like response is specific to carbon metabolism and mutation of genes encoding key steps of gluconeogenesis; the glyoxylate cycle and β-oxidation of fatty acids attenuate virulence to a greater or lesser degree [21], [22]. Single cell GFP reporters confirm induction of genes such as phosphoenolpyruvate carboxykinase (PCK1) and isocitrate lyase (ICL1) in macrophages and in tissues [21]. The regulatory networks that control expression of these genes differ markedly from the *S. cerevisiae* paradigms [23].

What carbon sources are most relevant in vivo? Some host niches are clearly glucose-deficient. Lactate, produced by tissues and by bacteria in the gut, is one potential carbon source. Fungi generally prefer glucose to the exclusion of any other carbon sources, but *C. albicans* cells metabolize glucose and lactate concurrently in at least some circumstances [24]. Cells grown on lactate have an altered cell wall; are more resistant to osmotic, envelope, and antifungal stresses; and are more adherent [25]. Lactate-grown cells elicit lower levels of proinflammatory cytokines from monocytic cells but, once phagoctyosed, actually do more damage to macrophages [26]. Thus, exposure to nonpreferred carbon sources benefits *C. albicans* in its interactions with macrophages. It is likely that this flexible organism finds and uses multiple carbon sources within the host.

We have proposed that amino acids are another relevant in vivo nutrient [27]. Phagocytosed cells induce the entire arginine biosynthetic pathway but no other amino acid synthetic genes [20], [28]. Surprisingly, expression results from exposure to moderate concentrations of ROS, rather than a lack of arginine. Induction is not seen when phagocytosed by ROS-deficient macrophages lacking the gp91 subunit of the phagocyte oxidase [28]. The significance of this connection between nutrients and oxidative stress is not clear.

## Intracellular Trafficking: Losing One's Way

We are beginning to understand the molecular interactions at the interface between host and pathogen cells that lead to endocytosis and the activation of responses in both cells. In contrast, the intracellular fate of *C. albicans* once phagocytosed remains a mystery. Early investigations came to contradictory conclusions about whether phagosome–lysosome fusion occurred, depending on the approach used. A more recent analysis using markers along the endocytic pathway during phagosome maturation demonstrated that intracellular trafficking of *C. albicans* is aberrant, but a definitive mapping of these events was obscured by heterogeneity in postendocytic events [29]. Relative to heat-killed controls, phagosomes with live *C. albicans* cells were associated with less filamentous actin and acquired late endosomal markers less robustly, including LAMP-1 and the vATPase proton pump. Even transient colocalization with late endosomal makers was rapidly lost and, in phagosomes containing filamentation-competent cells, replaced with elements normally associated with the ER membrane, such as calnexin. Live *C. albicans* appears to inhibit both lysosomal acidification and NO release [29]. We have also identified macrophage-like conditions in which *C. albicans* releases ammonia derived from amino acid degradation to raise extracellular pH [27], potentially synergizing with the vATPase defect to block acidification. On top of this, some phagocytosed *C. albicans* cells escape macrophages through a non-lytic route [30]; the underlying mechanisms, again, are not understood.

The take-home message is that the intracellular fate of live *C. albicans* differs markedly from that of killed cells, implying an active modulation by the fungal cell. At least some filamentous cells escape the normal phagocytic pathway to an “ER-like” compartment. Further study, particularly with more refined temporal resolution to reduce heterogeneity, is clearly needed to understand the intracellular fate of this organism.

## Morphogenesis: The Shape of What's to Come

**Figure 1:**
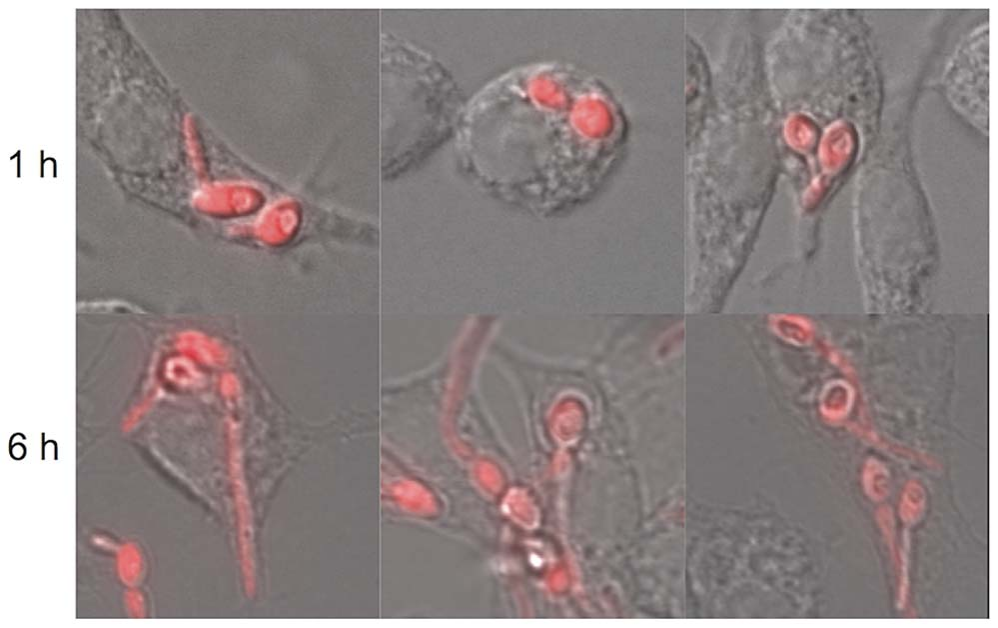
Morphogenesis of *C. albicans* within macrophages. *C. albicans* cells constitutively expressing yCherry were incubated for one hour (top) or six hours (bottom). Germination is apparent early and has disrupted macrophage structures by the later time point.

Morphological differentiation is a central theme of fungal pathogenesis, from the multicellular development of *Aspergillus* species to the hyphal-to-yeast transition in the host of the dimorphic fungi, such as *Histoplasma* and *Coccidioides*. *C. albicans* yeast cells germinate within the macrophage, forming hyphae that penetrate and kill the phagocyte, a remarkable aspect of this interaction (Figure 1). Yeast, hyphae, and pseudohyphae can be seen simultaneously in tissues, and this reversible switch is a core virulence trait, as mutants locked in one form are avirulent [31], [32]. The regulation of hyphal morphogenesis is extremely complex, with multiple inducing signals, including neutral pH, elevated CO_2_, serum, physiological temperatures, and N-acetylglucosamine, feeding into signaling pathways that culminate in dozens of transcription factors (reviewed in [33]).

It is not clear why *C. albicans* switches to the hyphal form within the macrophage. The intracellular environment should be acidic and carbon-poor, which are conditions inhibitory to filamentous growth. The aberrant trafficking discussed above may put *C. albicans* in more conducive conditions, but what actually induces the transition is unknown. Two possibilities impinge on amino acid metabolism: Mutants lacking *arg4*Δ form hyphae less well than wild-type strains within macrophages, and it has been proposed that *C. albicans* cells synthesize and then degrade arginine for the purpose of generating CO_2_ [34], which is consistent with our demonstration that this pathway is induced [28]. More broadly, catabolism of amino acids as a carbon source in vitro releases ammonia as a byproduct, resulting in a dramatic rise in the extracellular pH, and we proposed that this occurs when in the macrophage [27]. ROS may also induce the hyphal switch, as *cap1*Δ mutants fail to filament after phagocytosis [14]. Either or both of these phenomena could create hyphal-inducing conditions in the macrophage.

**Figure 2:**
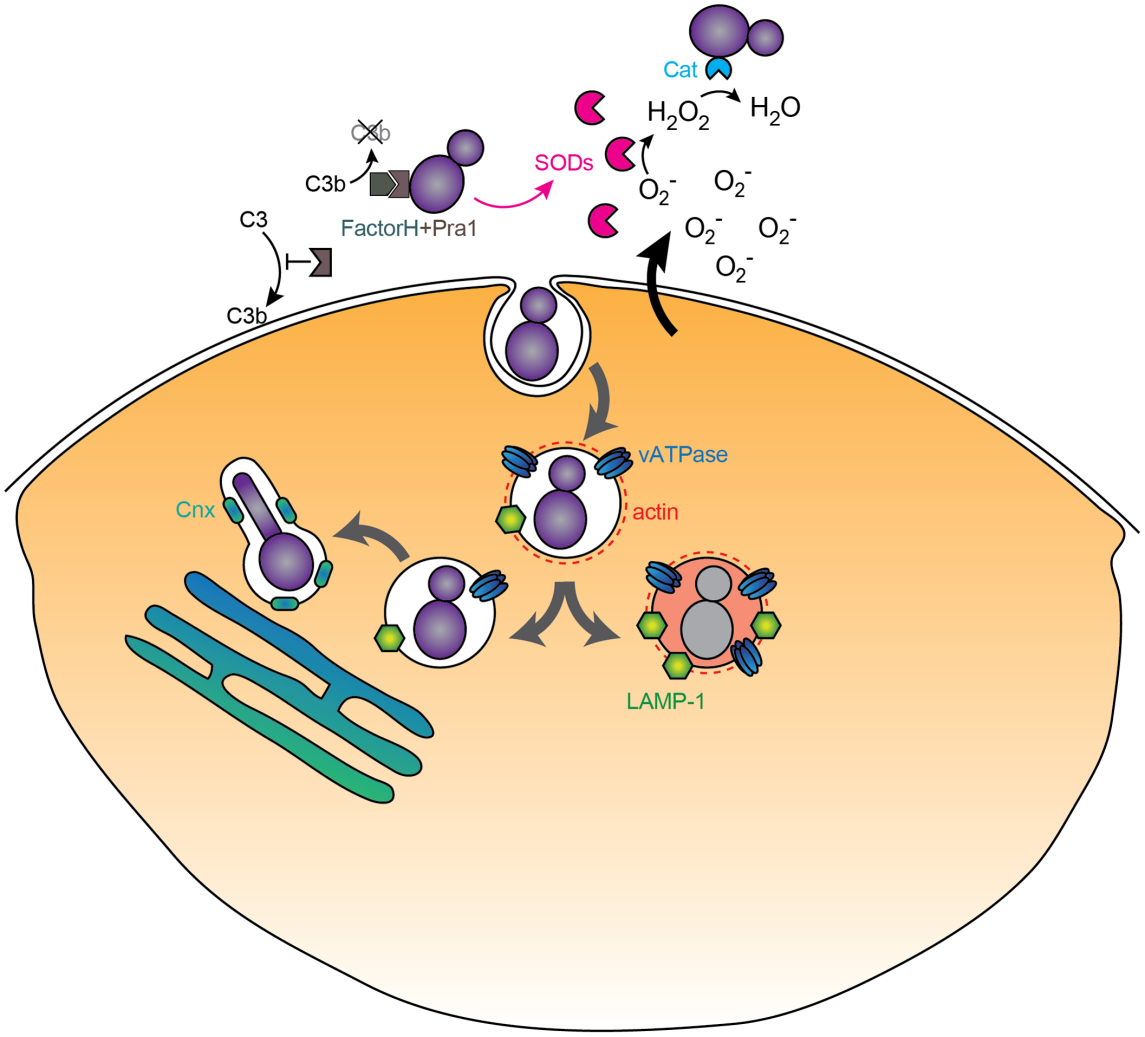
Immunomodulatory activities of *C. albicans*. Extracellular *C. albicans* inhibits complement deposition and detoxifies ROS via secreted mediators (SODs, catalase, Pra1), while phagocytosed cells proceed along an altered trafficking pathway to end up in an ER-associated compartment characterized by the membrane calnexin (Cnx) and a loss of LAMP-1, peripheral actin, and vATPase.

In summary, the *C. albicans*–macrophage model is a dynamic interaction in which this opportunistic pathogen employs multiple avenues to blunt the antimicrobial activity of the phagocyte by inhibiting recognition, trafficking, and effector release (Figure 2), while overcoming several important stresses. While much is left to learn, this is an important model system for understanding host–pathogen interactions.
